# Precipitating factors of diabetic ketoacidosis in type 1 diabetes patients at a tertiary hospital: a cross-sectional study with a two-time-period comparison

**DOI:** 10.20945/2359-3997000000480

**Published:** 2022-06-02

**Authors:** Laura Emanuelle da Rosa Carlos Monteiro, Sheila Piccoli Garcia, Leonardo Grabinski Bottino, Julia Luchese Custodio, Gabriela Heiden Telo, Beatriz D. Schaan

**Affiliations:** 1 Universidade Federal do Rio Grande do Sul Faculdade de Medicina Porto Alegre RS Brasil Faculdade de Medicina, Universidade Federal do Rio Grande do Sul, Porto Alegre, RS, Brasil; 2 Universidade Federal do Rio Grande do Sul Programa de Pós-graduação em Endocrinologia Porto Alegre RS Brasil Programa de Pós-graduação em Endocrinologia, Universidade Federal do Rio Grande do Sul, Porto Alegre, RS, Brasil; 3 Pontifícia Universidade Católica do Rio Grande do Sul Programa de Pós-Graduação em Medicina e Ciências da Saúde Porto Alegre RS Brasil Programa de Pós-Graduação em Medicina e Ciências da Saúde, Pontifícia Universidade Católica do Rio Grande do Sul, Porto Alegre, RS, Brasil; 4 Pontifícia Universidade Católica do Rio Grande do Sul Faculdade de Medicina Porto Alegre RS Brasil Faculdade de Medicina, Pontifícia Universidade Católica do Rio Grande do Sul, Porto Alegre, RS, Brasil; 5 Universidade Federal do Rio Grande do Sul Hospital de Clínicas de Porto Alegre Divisão de Endocrinologia Porto Alegre RS Brasil Divisão de Endocrinologia, Hospital de Clínicas de Porto Alegre, Universidade Federal do Rio Grande do Sul, Porto Alegre, RS, Brasil; 6 Conselho Nacional de Desenvolvimento Científico e Tecnológico Instituto Nacional de Ciência e Tecnologia para Avaliação de Tecnologias em Saúde Porto Alegre RS Brasil Instituto Nacional de Ciência e Tecnologia para Avaliação de Tecnologias em Saúde – Conselho Nacional de Desenvolvimento Científico e Tecnológico (CNPq), Porto Alegre, RS, Brasil

**Keywords:** Diabetic ketoacidosis, precipitating factors, type 1 diabetes mellitus

## Abstract

**Objective:**

To evaluate the precipitating factors of diabetic ketoacidosis (DKA) in patients with type 1 diabetes hospitalized through the emergency department of a tertiary hospital.

**Materials and methods:**

Individuals with type 1 diabetes hospitalized for DKA from January 2005 to March 2010 (first period [P1], n = 75) and from April 2010 to January 2017 (second period [P2], n = 97) were identified through a query of electronic medical records. Data were collected by reviewing medical records. Only the first hospitalization of each participant in each period was included.

**Results:**

In P2, 44 patients (45.4%) were women, mean age was 26.2 ^±^ 14.5 years, and 74 patients (76.3%) had a previous diagnosis of type 1 diabetes. Only 1 patient had glycated haemoglobin (HbA1c) below 64 mmol/mol (8.0%). Most patients (62.2%) had had a previous episode of DKA. In P1, non-adherence was the main cause of DKA (38.7%), followed by infection (24.0%). In P2, these rates were 34.0% and 24.7%, respectively; no statistical difference was observed between the two study periods (p = 0.790).

**Conclusion:**

Over time, non-adherence remained the main precipitating factor of DKA, followed by infection, and no significant difference was observed between the two study periods. Elevated HbA1c, outside the therapeutic range, indicates suboptimal diabetes care and may explain, at least in part, poor adherence as a precipitating factor of decompensation. Health strategies, such as improved self-management of type 1 diabetes, may contribute to a future reduction in DKA episodes.

## INTRODUCTION

Diabetic ketoacidosis (DKA) is a potentially life-threatening complication of type 1 diabetes. Its prevalence has increased over the past two decades, with approximately 50 to 100 events per 1,000 adult patients with type 1 diabetes (1–3). The overall mortality from paediatric DKA in the United States is approximately 0.5%, and the number of hospitalizations for DKA among diabetic adults aged ≥18 years was 168 000 (7.7 per 1000 diabetic persons) in 2014 (4). The management of an episode of DKA also imposes a high cost on a country's health system – estimated at € 2064 in the United Kingdom, for example – and the precipitating cause could be an important determinant of cost (5).

The main known DKA precipitating factors are newly diagnosed diabetes, infection, poor adherence to treatment, and problems with interruption of insulin delivery (6,7). Approximately one-third of children with type 1 diabetes present with DKA on diagnosis (8). In developed nations, poor adherence to treatment is the leading cause of DKA, followed by infection and newly diagnosed diabetes, whereas infection and limited access to health care are the most prevalent causes in developing countries (9).

In 2011, we published an article describing the main triggers of DKA in patients with type 1 diabetes at a public tertiary hospital from January 2005 to March 2010, which indicated poor adherence as the main cause of this acute complication, followed by infection and newly diagnosed diabetes (6). The objective of the current study was to determine if there were changes in the precipitating factors of patients presenting to the emergency department with DKA in the same hospital over time.

## MATERIALS AND METHODS

This report followed the Strengthening the Reporting of Observational Studies in Epidemiology (STROBE) guidelines (10).

### Study design

We developed a cross-sectional study to establish the most common precipitating factors of DKA in patients with type 1 diabetes in two different time periods at a public tertiary hospital.

### Setting

The study analysed patients from a tertiary university hospital located in southern Brazil, a middle-income country. The data were divided into two time periods for comparison: period 1 (P1), from January 2005 to March 2010; and period 2 (P2), from April 2010 to January 2017. After identifying the patients who met the criteria for DKA, their electronic medical records were analysed for precipitating factors of DKA and other associated factors.

### Study population

Patients were identified through a query of electronic medical records. All patients presenting to the emergency department who had blood collected for arterial blood gas analysis and measurement of serum or urine ketones and blood glucose had their medical records reviewed. We retrospectively selected patients with type 1 diabetes who met the diagnostic criteria for DKA, defined by:(11)

- Capillary or plasma glucose > 250 mg/dL (13.0 mmol/L);
- Metabolic acidosis (pH < 7.30 or serum bicarbonate < 18 mmol/L);
- Ketosis (detectable ketone bodies in serum or urine).

When patients had been hospitalized more than once during the study periods, only the first hospitalization was counted for each patient in the period. However, the periods were considered independently, i.e., patients could be included in both periods, but only their first hospitalization in each period was considered.

Considering that approximately one third of people with type 1 diabetes have DKA at diagnosis, for a better assessment of precipitating causes of this complication, we performed the analyses in two ways. First, evaluating all patients diagnosed with DKA, including both patients with a previous diagnosis of type 1 diabetes and patients who received type 1 diabetes diagnosis at the emergency department. Secondly, we evaluated the precipitating causes of DKA only in patients with previously instituted treatment, that is, with a previous diagnosis of type 1 diabetes.

### Variables

The precipitating factors were infection, treatment poor adherence, DKA associated with the diagnosis of type 1 diabetes (initial diagnosis), recreational drug use, and others (defined as those not belonging to any of the previous groups and, therefore, analysed individually). As in the previous study, treatment poor adherence was considered a precipitating factor if there was non-adherence to insulin therapy or dietary abuse (without proper correction with bolus insulin) excluding any other clearly identifiable precipitating factor (6). The precipitating factor of DKA was defined by the evaluation of physicians or endocrinologists during hospitalization.

Other variables were collected for each patient: age; sex; skin colour; occupation; weight and height; previous diagnosis of type 1 diabetes; duration of diabetes; treatment-related data, such as medications, doses, and methods of insulin administration; chronic complications of diabetes; hypertension; psychiatric disorders (diagnosed by mental health specialists); alcohol consumption; drug addiction (cocaine/crack cocaine, marihuana, alcohol abuse, other); smoking; previous DKA; number of absences and attendances at medical and nutritional scheduled appointments; previous levels of glycated haemoglobin (HbA1c); precipitating factor and outcome of DKA; and laboratory tests on arrival at the emergency department (plasma glucose, arterial pH, serum bicarbonate, creatinine, potassium, sodium, and serum and urine ketones). Other laboratory and radiological investigations performed for precipitating factors were also analysed, including chest and sinus radiographs and urine and blood cultures.

### Data sources/Measurements

HbA1c was measured by high-performance liquid chromatography on the Variant II Turbo analyser (BioRad, Hercules, CA, USA) using the cation-exchange chromatographic method. Plasma glucose was determined by an enzymatic UV method (hexokinase method), serum creatinine by the kinetic Jaffé colorimetric method, and serum bicarbonate by the Cobas c702 enzymatic assay (Roche, Naka, Japan). Ketones were measured by reagent strip testing with sodium nitroprusside. Serum pH was determined in arterial blood with the ABL-800 Flex gas analyser (Radiometer, Copenhagen, Denmark).

### Sample size

The number of patients presenting to the emergency department who met the inclusion criteria during the study period determined the sample size (n = 97).

### Statistical analysis

Descriptive data are expressed as number (%), mean and standard deviation (SD), or median and interquartile range (25th-75th percentiles [P25-P75]). The t-test was used for continuous variables, and the chi-square test for categorical variables. Data were analysed using SPSS, version 20.0, and the level of significance was set at p < 0.05 for all analyses.

### Ethics

The study was approved by the institution's Research Ethics Committee (protocol no. CAEE 67551415.6.0000.5327) and was performed according to the ethical principles established by the Declaration of Helsinki.

## RESULTS

**Figure 1:**
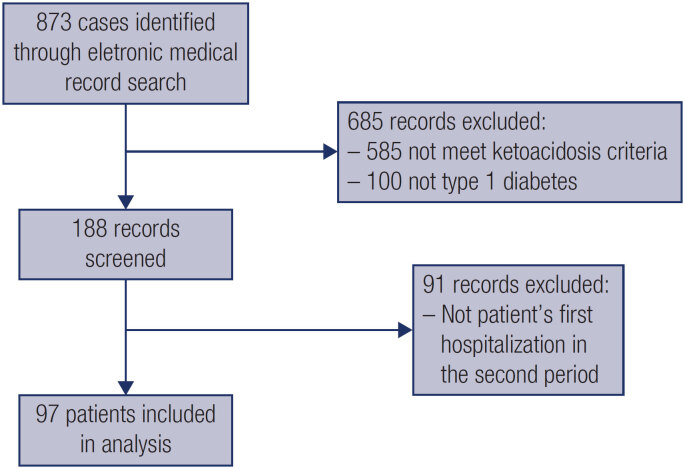
Flow diagram of cases included in the second period analysis. During the electronic medical record search, 873 potential cases of diabetic ketoacidosis (DKA) were identified. After reviewing the medical records, 685 records were initially excluded. Then, only the first DKA hospitalization of each patient was included, resulting in 97 patients included.

During the electronic medical record search, 873 potential cases of DKA were identified. After completing the review of medical records, 585 records were excluded for not meeting the criteria for DKA and 100 records were excluded due to other type of diabetes (rather than type 1). After selecting all confirmed cases of DKA in patients with type 1 diabetes, only the first DKA hospitalization of each patient was included, leading to the exclusion of 91 records (Figure 1).

A total of 97 patients were included in the study. The clinical characteristics of patients are described in Table 1. Briefly, 44 (45.4%) were women, mean (SD) age was 26.2 (14.6) years, and 78 (80.4%) were white. Forty-three (46.2%) were professionally active and 32 (34.4%) were students. Seventy-four patients (76.3%) had a previous diagnosis of diabetes on admission, and the remaining 23 (23.7%) were newly diagnosed with diabetes. The median duration of diabetes was 10.0 years (P25-P75, 5-19 years), and 46 patients (62.2%) had already had a previous episode of DKA. Eighteen patients (24.3%) were identified as having a psychiatric disorder, such as anxiety (n = 2), depression or bipolar mood disorder (n = 8), schizophrenia (n = 3) and drug addiction (n = 5).

**Table 1:** Clinical characteristics of all patients included (N = 97) in the second period analysis

Characteristics	N = 97
Age (years)	26.2 ± 14.6
Previously diagnosed type 1 diabetes (%)	74 (76.3)
Women (%)	44 (45.4)
White (%)	78 (80.4)
Actively employed (%)	43 (46.2)

Data presented as number (%) or mean ± standard deviation.

**Figure 2:**
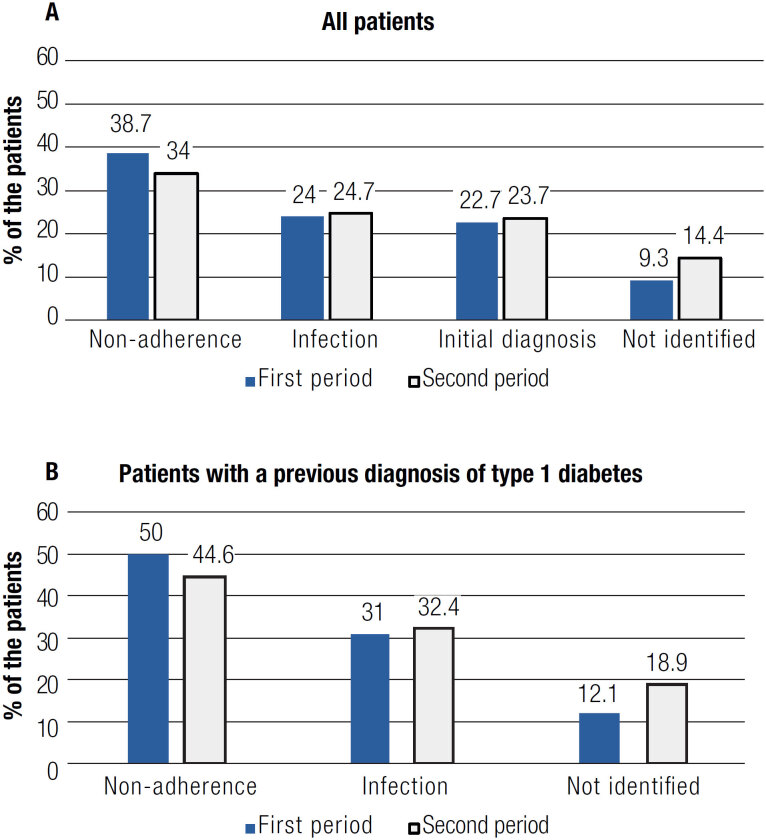
Causes of diabetic ketoacidosis in both study populations, diagnosed in the first period (P1) and second period (P2). **Panel A:** All patients included. In P1 (n = 75), poor adherence accounted for 38.7%; infection, 24.0%; initial diagnosis, 22.7%; and not identified, 9.3%. In P2 (n = 97), poor adherence accounted for 34.0%; infection, 24.7%; initial diagnosis, 23.7%; and not identified, 14.4% (p = 0.790). **Panel B:** Only patients with a previous diagnosis of type 1 diabetes included. In P1 (n = 58), poor adherence accounted for 50.0%; infection, 31.0%; and not identified, 12.1%. In P2 (n = 74), poor adherence accounted for 44.6%; infection, 32.4%; and not identified, 18.9% (p = 0.642).

The main triggering factors identified were poor adherence to treatment (n = 33, 34.0%), infection (n = 24, 24.7%), and initial diagnosis (n = 23, 23.7%). In 14 patients (14.4%), the precipitating factor could not be identified (Figure 2, panel A). Clinical characteristics among individuals with DKA triggered by infection or poor adherence are shown in Table 2. There is no significant difference between groups considering the two main causes. Urinary tract infection was the most common infection, detected in 11 cases (11.8%), followed by respiratory tract infection (n=5, 5.4%) and erysipelas (n = 5, 5.4%).

**Table 2:** Clinical characteristics of patients in the second period (P2) according to the two main precipitating factors of DKA

Characteristics		Infection (N = 24)	Non-adherence (N = 31)		
Age (years)		31 ± 11.1	26.6 ± 12.2		
Women (%)		13 (54.2)	17 (54.8)		
White (%)		20 (83.3)	24 (77.4)		
Duration of diabetes (years)		14 (8-22.5)	10.5 (3.8-17.5)		
Previous DKA (%)		15 (62.5)	16 (51.6)		
HbA1c in the last 3 months (%)		12.3 ± 2.3	13.1 ± 2.8		
Diabetes complication (%)			
	Neuropathy	4 (16.7)	1 (3.2)		
	Diabetes renal disease	8 (33.3)	5 (16.1)		
	Retinopathy	5 (20.8)	4 (12.9)		
	Ischemic heart disease	4 (16.7)	3 (9.7)		
	Cerebrovascular disease	0 (0)	2 (6.5)		
Comorbidities (%)			
	Systemic arterial hypertension	4 (16.7)	7 (22.6)		
	Psychiatric disorder	5 (20.8)	7 (22.6)		
	Drug addiction	2 (8.3)	0 (0)		
	Social problems	2 (8.3)	7 (22.6)		

Data presented as number (%), mean ± standard deviation, or median (P25-P75). DKA: diabetic ketoacidosis. HbA1c: glycated haemoglobin.

Considering patients with previous measurement of HbA1c recorded in the medical records (n = 33), only one (1.0%) had HbA1c levels below 8.0% (64 mmol/mol). Regarding the treatment, only one patient was treated with insulin infusion pump, and only one patient used insulin pens. None of the patients were using sodium-glucose cotransporter-2 (SGLT2) inhibitors.

Of the 74 patients with a previous diagnosis of type 1 diabetes, 16 (21.6%) had systemic arterial hypertension, 12 (23.5%) had some degree of diabetic retinopathy, 18 (24.3%) had diabetic renal disease, 8 (10.8%) had diabetic neuropathy, and 7 (9.5%) had ischemic heart disease. The rates of diabetes complications and comorbidities of P2 patients were similar to those of P1 patients (Table 3). P1 patients were slightly younger than P2 patients (p = 0.037).

**Table 3:** Clinical characteristics of patients with a previous diagnosis of type 1 diabetes in the first period (P1) and second period (P2)

Characteristics		P1 (N = 58)	P2 (N = 74)	p
Age (years)		23.9 ± 12.3	28.6 ± 13.0	0.037
Women (%)		29 (50.0)	37 (50.0)	1.0
White (%)		46 (79.3)	61 (82.4)	0.896
Duration of diabetes (years)		8.5 (5-13)	10 (5-19)	0.124
Previous DKA (%)		34 (59.0)	46 (62.2)	0.906
HbA1c in the last 3 months (%)		11.6 ± 2.7	12.5 ± 3.4	0.184
HbA1c in the last 3 months (mmol/mol)		103 ± 29.5	113 ± 27.2	0.184
Diabetes complication (%)				
	Neuropathy	6 (10.3)	8 (10.8)	1.00
	Diabetes renal disease	14 (24.1)	18 (24.3)	1.00
	Retinopathy	7 (12.1)	12 (16.2)	0.620
	Ischemic heart disease	1 (1.75)	7 (9.5)	0.078
	Cerebrovascular disease	2 (3.4)	4 (5.4)	0.694
Comorbidities (%)				
	Systemic arterial hypertension	9 (15.5)	16 (21.6)	0.503
	Psychiatric disorder	15 (25.8)	18 (24.3)	0.537
	Drug addiction	10 (10.3)	5 (6.8)	0.448
	Social problems	9 (15.5)	10 (13.5)	0.805

Data presented as number (%), mean ± standard deviation, or median (P25-P75). DKA: diabetic ketoacidosis. HbA1c: glycated haemoglobin.

In P1, poor adherence was the main cause of DKA (38.7%), followed by infection (24.0%). No significant difference was observed between the two study periods (p=0.790). When only patients who had a previous diagnosis of diabetes were analysed, poor adherence to treatment accounted for 44.6% of cases (Figure 2, panel B). Compared with the first study period (up to 2010), when low adherence was identified in 50.0% of cases, there was only a minimal reduction in the number of noncompliant patients, with no statistically significant difference between the two study periods (p = 0.642).

## DISCUSSION

Diabetic ketoacidosis is a potentially life-threatening but preventable complication of diabetes and the leading cause of death in children and young adults with type 1 diabetes (12). Better understanding of the precipitating factors of DKA may facilitate the targeting of prevention efforts. However, there is scientific uncertainty about what is actually the most common precipitating factor. In our study, no statistical difference was observed between the two study periods. In the first-period analysis, covering the years from 2005 to 2010, poor adherence was the main cause of DKA, accounting for almost 40% of all cases, followed by infection (24%). In the second-period analysis, covering the years from 2010 to 2017, no changes were observed in the main causes of DKA, with non-adherence to treatment remaining the main precipitating factor. When only patients with a previous diagnosis of type 1 diabetes were evaluated, there was also no change in the distribution of the causes of DKA: rates were similar between the two study periods, with poor adherence to treatment as the main triggering factor for this acute complication of diabetes. The percentage of patients who did not have their DKA precipitating factor identified increased from 9.3% in the first period to 14.4% in the second period. Difficulty in identifying the precipitating factor of DKA has also been described in previous studies with a retrospective design (7,13,14) including the previous study conducted in the same hospital (6). Moreover, incomplete data are not uncommon under such study design, as data entry in electronic medical records is often considered both burdensome and time-consuming by physicians (15).

Poor adherence to treatment, especially insulin omission, has been widely reported as an important precipitating factor of DKA (16,17). Because it is a modifiable and preventable factor, clarifying and promoting adherence to treatment may reduce morbidity and mortality as well as the costs of diabetes care (18,19). This trigger, which is also involved in other outcomes such as chronic complications of diabetes, may be prevented through education of patients at increased risk of DKA, including strategies such as family counselling, patients’ education for self-management of their disease, and improved follow-up (20,21). Unfortunately, although diabetes care in our institution is provided by a multidisciplinary team (dietitians, nurses, psychologists, and endocrinologists) and educational initiatives are offered, individually or in groups, to most of our patients with diabetes, the impact on the outcomes measured here was null. This information should be used to develop new approaches to delivery diabetes self-management in order to have an actual impact on our patients in daily practice. Comprehensive strategies and innovative clinical models are needed to engage patients in their own diabetes care by addressing barriers to ideal management and, consequently, improving adherence to treatment; supported by telemedicine, for example (22).

Infection remained an important cause of DKA in the present study. This triggering factor appears to be more closely associated with older age and most commonly affects the lungs and urinary tract (23). This is particularly important considering a previous report in the literature of a significant association of infection with increased case-fatality rate of DKA, which indicates that more actions aimed at prompt identification and treatment of infection may be needed (24).

On admission, 23 patients (23.7%) were newly diagnosed with type 1 diabetes, which suggests that a significant proportion of cases of DKA are diagnosed at the initial presentation of the disease. Therefore, programs to improve the understanding of the classic symptoms of type 1 diabetes would increase early diagnosis and prevent acute metabolic disorders at presentation. This action may reduce the number of diagnoses of DKA in children at the initial presentation of type 1 diabetes, which is what occurs in centres with a larger number of cases of type 1 diabetes (4,8,25).

In the present study, nearly all patients had poor glycaemic control prior to hospitalization and had a significantly prevalence of psychiatric disorders (24%). These data are in accordance with previous studies correlating DKA events with elevated HbA1c and poor glycaemic control (1,26,27), and psychiatric disorders (28). Other factors commonly correlated with DKA events are lower socioeconomic status (27,28) and female sex (1,27). Most of our patients were male, and their socioeconomic status was not assessed, thus precluding further analysis of these data. The same rationale discussed above, regarding the rethinking of diabetes care including psychologic and/or psychiatric follow up, would probably also result in a larger number of patients with better metabolic control, and consequently lower DKA events (22).

Some limitations of our study, such as including only the first hospitalization of the same patient who had more than one admission for DKA, may have interfered with the interpretation of the results, since some patients had several hospitalizations for DKA. In addition, all medical records reviewed were exclusively from patients receiving care through the public health system, who are often of low socioeconomic status and more susceptible to social problems that may interfere with the control of chronic diseases and increase the risk of DKA (25). This was a retrospective cross-sectional study and, therefore, subject to known methodological biases. All information was collected only by medical record review, thus preventing the analysis of some data that were not described in all records. Nevertheless, to our knowledge, this study is one of the first in Latin America to estimate the number of DKA cases at a tertiary hospital in two different time periods, as well as to investigate possible identifiable precipitating factors over time.

In conclusions, after almost a decade, poor adherence to treatment remains the most common precipitating factor of DKA in our population. Outpatient follow-up, diabetes care programs, and implementation of initiatives to engage patients in diabetes self-management education and adherence to self-care may be effective strategies to reduce the number of hospitalizations for DKA, thus improving the overall quality of life of patients with type 1 diabetes.
